# Handling of Radical Prostatectomy Specimens: Total Embedding with Large-Format Histology

**DOI:** 10.1155/2012/932784

**Published:** 2012-07-10

**Authors:** Rodolfo Montironi, Antonio Lopez Beltran, Roberta Mazzucchelli, Liang Cheng, Marina Scarpelli

**Affiliations:** ^1^Section of Pathological Anatomy, School of Medicine, United Hospitals, Polytechnic University of the Marche Region, 60126 Ancona, Italy; ^2^Department of Pathology, Reina Sofia University Hospital and Faculty of Medicine, 14004 Cordoba, Spain; ^3^Department of Pathology and Laboratory Medicine, Indiana University School of Medicine, Indianapolis, IN 46202, USA

## Abstract

A problem when handling radical prostatectomy specimens (RPS) is that cancer is often not visible at gross examination, and the tumor extent is always underestimated by the naked eye. The challenge is increased further by the fact that prostate cancer is a notoriously multifocal and heterogeneous tumor. For the pathologist, the safest method to avoid undersampling of cancer is evidently that the entire prostate is submitted. Even though whole mounts of sections from RPS appear not to be superior to sections from standard blocks in detecting adverse pathological features, their use has the great advantage of displaying the architecture of the prostate and the identification and location of tumour nodules more clearly, with particular reference to the index tumour; further, it is easier to compare the pathological findings with those obtained from digital rectal examination (DRE), transrectal ultrasound (TRUS), and prostate biopsies. We are in favour of complete sampling of the RPS examined with the whole mount technique. There are reasons in favour and a few drawbacks. Its implementation does not require an additional amount of work from the technicians' side. It gives further clinical significance to our work of uropathologists.

## 1. Introduction

Handling of radical prostatectomy specimens is a challenging task for the pathologist. The prostate undergoes faster autolysis than most other organs, prostate cancer is notoriously difficult to identify with the naked eye, the tumors are smaller but yet more multifocal than most other clinically diagnosed cancers and prostate cancer is very heterogeneous, both morphologically and genetically. Thus, these specimens need to be handled with great care and according to standardized protocols to enable accurate assessment of grade and stage [1].

The aim of this contribution is to briefly review the current literature on complete versus partial sampling of radical prostatectomy specimens and on whole-mount versus standard sections. Special reference is made to the International Society of Urological Pathology (ISUP) consensus conference on handling and staging of radical prostatectomy specimens [2]. A final section of this paper is dedicated to the Ancona protocol based on the complete sampling of the surgical specimens with whole-mount sections [3].

## 2. Total versus Partial Embedding

A problem when handling radical prostatectomy specimens is that cancer is often not visible at gross examination, and the tumor extent is always underestimated by the naked eye. The challenge is increased further by the fact that prostate cancer is a notoriously multifocal and heterogeneous tumor. For the pathologist, the safest method to avoid undersampling of cancer is evidently that the entire prostate is submitted. In some institutions, partial sampling is practiced. This requires that the pathologist adheres to a strict protocol, which may be somewhat cumbersome [2, 4, 5].

In 1994, a report on how prostate specimens were examined by American pathologists showed that only 12% of pathologists embedded the entire prostate [6]. Since then the proportion of laboratories that use partial embedding has decreased. In a recent ENUP survey among 217 European pathologists from 15 countries, only 10.8% used partial embedding routinely [7]. In some European countries total embedding is even mandatory, according to national guidelines.

The recent study by Dr. Vainer et al. analyzes 238 radical prostatectomy specimens (RPS) to determine whether significant prognostic information is lost when a partial sampling approach with standard cassettes is adopted, compared with total embedding [8]. In their study, upon arriving at the Pathology Department, the prostate is partly divided by a cut in the mid-sagittal plane through the anterior surface, separating the two lobes for optimal fixation. The gland is then fixed for an additional 20 hours in formic acid and 24 hours in 4% buffered formalin. The gross examination includes measurement in three dimensions, weighing the prostate after removal of the seminal vesicles, and separating the left from the right lobe after inking the anterior and the posterior halves with two different colours. Apical and basal slices of 5–10 mm, depending on the total size of the RPS, are cut horizontally, subsequently sliced parasagittally, and placed in cassettes with often more than one section per cassette. The remaining part of the prostate is cut horizontally in approximately 3-mm thick slices and placed in standard cassettes, ensuring laterality. Large slices are divided to fit standard cassettes. Finally, sections from the seminal vesicles (as a minimum the apex and a cross-section) are embedded. Postfixation in 4% formalin and embedding in paraffin are followed by 4-*μ*m sectioning and staining with haematoxylin and eosin (no. of cassettes/total slides: 18 to 76). For the purpose of the study, glass slides from every second horizontal slice are withheld (no. of slides initially removed: 3 to 26, i.e., 29.9%). The remaining slides are evaluated microscopically.

According to this group of researchers, such an approach decreases the laboratory workload by 30%, and at the same time little information is lost with this procedure, overlooking features significant for the postoperative treatment in only 1.2%. They conclude that partial embedding is acceptable for valid histopathological assessment.

The findings reported by Dr. Vainer et al. [8]are slightly better than those reported by others. Hall et al. [4] showed that by submitting only gross stage B cancer along with standard sections of the proximal and distal margins, the base of seminal vesicles, and the most apical section (next to distal margin), 96% of positive surgical margins and 91% of instances of extraprostatic extension were detected, as compared with identification by complete microscopic examination. In the study by Cohen et al. [9] involving patients with clinical stage B carcinoma, each gland was serially sectioned with sections mounted whole on oversized glass slides. Using only alternate sections, there was a 15% false-negative rate for extraprostatic extension. In a study by Sehdev et al. [5], cT1c tumours with one or more adverse pathological findings, such as Gleason score 7 or more, positive, margins and extraprostatic extension, were compared using ten different sampling techniques. The optimal method consisted of embedding every posterior section and one mid-anterior section from the right and left sides of the gland. If either of the anterior sections had sizable tumour, all anterior slices were blocked in a second step. This method detected 98% of tumours with Gleason score 7 or more, 100% of positive margins, and 96% of cases with extraprostatic extension, through examination of a mean number of 27 slides. It was also shown that sampling of sections ipsilateral to a previously positive needle biopsy detected 92% of Gleason score 7 or greater cancers, 93% of positive margins and 85% instances of extraprostatic extension, from a mean number of 17 slides.

## 3. Whole-Mount versus Standard Sections

Radical prostatectomy specimens may be processed as either whole-mount or standard sections. Disadvantages with whole-mount sections that include recuts are more difficult to make and it is more expensive and difficult to perform immunohistochemistry. Tissue microarrays can be constructed from whole-mounts for immunohistochemistry, but this technique damages the paraffin blocks and it is a time-consuming process to set up a tissue microarray experiment on prostate cancer. Moreover, whole-mount sections do not fit into standard slide holders for slide collections and standard slide archives. However, whole-mount sections give the pathologist a better overview and the identification of multiple separate tumor foci is facilitated. Laboratory technicians who are trained to cut whole-mounts may find them less time-consuming than cutting multiple small blocks. Thus, the choice between whole-mounts versus standard sections is entirely up to the individual laboratory and should not be standardized [1].

## 4. 2009 International Society of Urological Pathology Survey and Consensus Conference

In order to identify the methods most commonly employed by urological pathologists worldwide, a web-based survey on handling and reporting of radical prostatectomy specimens was distributed to 255 members of the International Society of Urological Pathology. The International Society of Urological Pathology survey was followed up with a consensus conference held in conjunction with the 2009 Annual Scientific Meeting of the United States and Canadian Academy of Pathology held in Boston, Massachusetts. The aim was to obtain consensus relating to the handling and reporting of radical prostatectomy specimens. Those who completed the electronic survey were invited to attend the consensus conference, which was held on 8 March [2].

Many recommendations of this consensus conference have already been incorporated into international guidelines, including the recent College of American Pathologists protocol and checklist for reporting adenocarcinoma of the prostate and the structured reporting protocol for prostatic carcinoma from the Royal College of Pathologists of Australasia [10, 11].

In response to the question relating to how much of the prostate should be blocked, >60% of conference participants supported complete embedding, whereas >60% also supported partial embedding. This apparent contradiction arose as several respondents selected both options depending on the situation. In view of this, it was concluded that both methods were considered acceptable. Pathologists have to balance the extra expense and time involved in processing entire specimens against the risk of missing important prognostic parameters, and decide whether partial or complete embedding should be performed. There was consensus that if partial embedding is performed, a specific protocol should be followed and the methodology should be documented in the pathology report [2].

From the survey, a majority of respondents reported using standard blocks and only 16% reported the use of whole-mounts, for at least some slices. A minority reported using both methods. On discussion at the consensus conference it was considered that both standard blocks and whole-mounts were acceptable for examination of radical prostatectomy specimens, although no ballot was taken on this point [2].

## 5. Ancona Experience

**Figure 1:**
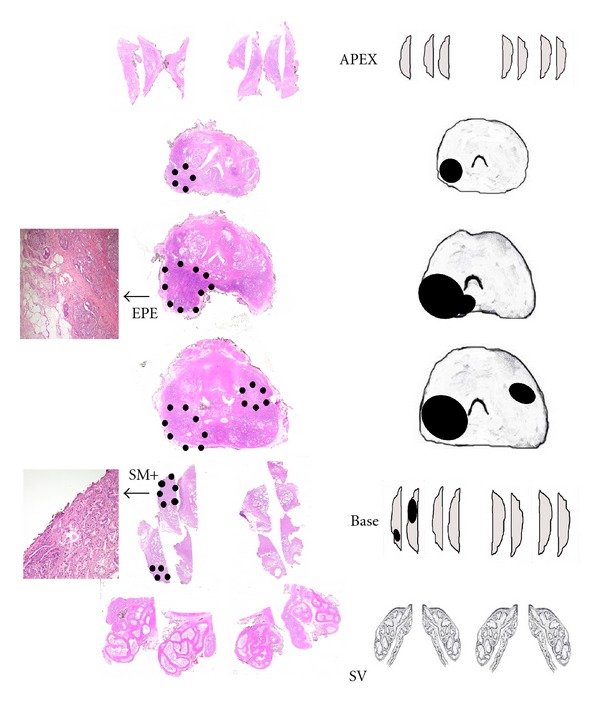
Complete sampling with the whole-mount technique of a prostate specimen. Hematoxylin and eosin-stained sections of prostate specimen are shown on the left and the corresponding mapping on the right. The dotted areas on the slides and the black areas of the map represent two prostatic cancer foci, the index tumour being present on the left of the slides. Extraprostatic extension (EPE) and positive surgical margin (SM+) are present in the posterolateral aspect of the body of the prostate and in one of the slides of the base (see details in the separate images) (SV: seminal vesicles).

In the last few years, 3,000 RPS have been totally embedded and examined with the whole-mount technique by one of our group (RM) at the Section of Pathological Anatomy of the Polytechnic University of the Marche Region and United Hospitals, Ancona, Italy (Figure 1).

The prostate is received fresh from the operating room. Its weight without the seminal vesicles and all three dimensions (apical to basal (vertical), left to right (transverse), and anterior to posterior (sagittal)) are recorded, the latter used for prostate volume calculation. To enhance fixation, 20 mL 4% buffered formalin is introduced into the prostate at multiple sites using a 23G needle. To ensure homogenous fixation the needle is inserted deeply and the solution injected while the needle is retracted slowly. The specimen is then covered with India ink and fixed for 24 hours in 4% neutral buffered formalin. After fixation, the apex and base (3 mm thick slices) are removed from each specimen and examined by the cone method. The prostate body is step-sectioned at 3 mm intervals perpendicular to the long axis (apical-basal) of the gland. For orientation a cut with a surgical blade is made in the right part of each prostate slice. The seminal vesicles are cut into two halves (sandwich method) and processed *in toto*. The cut specimens are postfixed for an additional 24 hours in 4% neutral buffered formalin and then dehydrated in graded alcohols, cleared in xylene, embedded in paraffin (the material is processed together with regular cassettes), and examined histologically as 5 *μ*m-thick whole-mount haematoxylin and eosin (H&E) stained sections [12].

The body of each prostate is represented with 3 to 6 whole-mount slides, whereas the apex, base, and seminal vesicles with 6 to 8 regular slides, totalling between 9–14 slides (in Dr. Vainer et al.'s study [8], up to 76 regular slides are needed to examine the whole prostate). The time needed to section each specimen with an ordinary delicatessen meat slicer is 15–20 minutes. The time taken by a technician to cut all the blocks of an individual case is 30–40 minutes. The time needed by the pathologist to report a case ranges from 40 to 60 minutes. Since the slides do not fit into the current staining machines, the slides are manually stained. The paraffin blocks and glass slides are stored in dedicated containers because of their large size. The comparison between Dr. Vainer et al.'s and our approach is presented in Table 1 [8].

Slides with substandard sections, however with cancer still evaluable, were observed in 7 cases (0,23% of RPS). Only in one case (0,03%) the quality was so poor that the features could not be evaluated. An individual block had to be serially sectioned to visualize the entire inked surface in 15 cases (0,5%). Immunohistochemistry (mainly the basal cell marker p63, racemase and chromogranin A) was done, always successfully, in 30 cases (1%), cutting from the whole-mount section the part to be evaluated in 28, and using the whole-mount section in the remaining two. A procedure was developed to search for residual cancer prostate cancer on pT0 radical prostatectomy after positive biopsy [13, 14]. When applied to 10 cases, a minute focus of cancer was successfully found in 8.

The complete set of slides of each case is examined macroscopically and then microscopically and information on morphological items with diagnostic and prognostic importance are gathered and interpreted in conjunction with clinical information and the macroscopic description of the specimen, including the following:

1. quality indicators of the surgical procedure: specimen integrity, including missing parts, capsular incision into tumour, and benign glands at the surgical margins;
2. type of surgical procedure applied, that is, nerve sparing, and previous surgical procedure, such as transurethral resection of the prostate;
3. presence of tissues other than prostate, that is, rectal wall;
4. morphologic prognostic and predictive features, such as Gleason score, stage, surgical margin status, and tumour volume;
5. comparison of pathological findings with digital rectal examination (DRE), transrectal ultrasound (TRUS), and prostate biopsies findings.

Even though whole-mounts of sections from RPS appear not to be superior to sections from standard blocks in detecting adverse pathological features [9], their use has the great advantage of displaying the architecture of the prostate and the identification and location of tumour nodules more clearly, with particular reference to the index tumour; further, it is easier to compare the pathological findings with those obtained from DRE, TRUS, and prostate biopsies. 

## 6. Conclusions

At the 2009 International Society of Urological Pathology consensus conference on handling and staging of radical prostatectomy specimens it was recommended that pathologists balance the expense and time involved in processing entire specimens against the risk of missing important prognostic parameters, and decide whether partial or complete embedding should be performed. A majority of respondents reported using standard blocks and only 16% reported the use of whole-mounts, for at least some slices.

We are in favour of complete sampling of the RPS examined with the whole-mount technique. There are reasons in favour and a few drawbacks. Its implementation does not require an additional amount of work from the technicians' side. It gives further clinical significance to our work of uropathologists [15]. In particular it gives us important pieces of information with paramount importance in relation to the definition of insignificant versus significant prostate cancer as well as to contemporary approaches in prostate cancer treatment, including active surveillance and focal therapy [16].

## Figures and Tables

**Figure 2:**
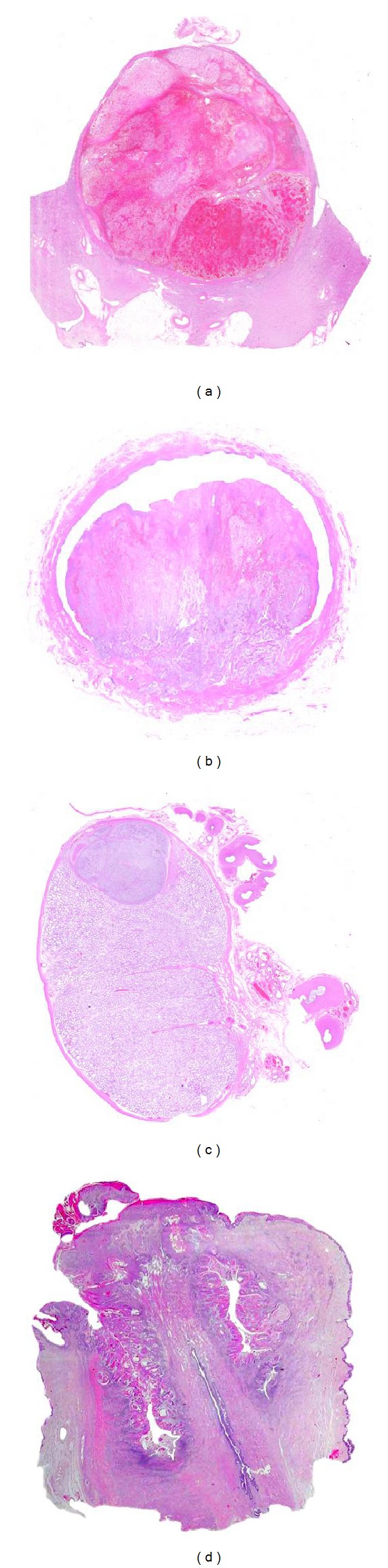
Examples of large-format histology of a kidney with clear cell renal cell carcinoma (a), of a urinary bladder with urothelial carcinoma (b), of a testis with seminoma (c) and a penis with squamous cell carcinoma (d).

**Table 1:** Comparison between Dr. Vainer et al.'s study [8] and Ancona experience [3].

Features	Dr. Vainer et al.'s study	Ancona experience
Prostate weight and size (and volume)	Yes (not mentioned)	Yes (yes)
Fixation enhancement	Separating the two lobes	Formalin injection
Inking of the surface	Two colours, anterior, and posterior halves	One colour; orientation with a cut on the right
Presectioning fixation (time)	Acid formic (20 h) and 4% buffered formalin (24 h)	4% buffered formalin (24 h)
Sectioning interval	Approximately 3 mm (Apex and base: 5–10 mm)	3 mm (Apex and base: 3 mm)
Subdivision of the slices of the prostate body	Yes, to fit standard cassettes	No (whole mounts)
Seminal vesicles	As a minimum the apex and a cross-section	Sandwich method (all included)
Postsectioning fixation (time)	4% buffered formalin (not mentioned)	4% buffered formalin (24 h)
No. of cassettes/total slides (% examined)	18–76 (70%)	9–14 (100%)
Processing	Not mentioned	As for regular size cassettes
Slide size (section thickness)	7.5 cm by 2.5 cm (4 *μ*m)	7.5 cm by 5.0 cm (5 *μ*m)
Slide staining procedure	Not mentioned	Manual

## Appendix

At the Section of Pathological Anatomy of the Polytechnic University of the Marche Region and United Hospitals, Ancona, Italy, a large-format histology is also used to evaluate tumors of the kidney, urinary bladder, testis, and penis.  Figure 2 shows examples of large-format histology of kidney with clear cell renal cell carcinoma (a), of urinary bladder with urothelial carcinoma (b), of testis with seminoma (c) and penis with squamous cell carcinoma (d).
